# Supplementary material for: Examining the role of Acinetobacter baumannii plasmid types in disseminating antimicrobial resistance
Source: NPJ Antimicrob Resist. 2024 Jan 5;2:1. doi: 10.1038/s44259-023-00019-y (PMC11702686; doi:10.1038/s44259-023-00019-y)
Supplement: Supplementary file 1 — Supplemental Material [file 44259_2023_19_MOESM1_ESM.pdf]

SUPPLEMENTARY MATERIAL

Supplementary Table 1: Available at https://doi.org/10.6084/m9.figshare.24076776

Supplementary Table 2: Available at https://doi.org/10.6084/m9.figshare.24076779

Supplementary Table 3. Properties of strains carrying pRAY* and its variants

Plasmid name	Strain name	Length (bp)	ST	Year	Isolation source	Country	Accession number
pA297-1	A297 (RUH875)	6078	1	1984	nr	Netherlands	KU869529
pD36-2	D36	6078	81	2008	wound	Australia	CP012954
pMRSN3527-6	MRSN 3527	6068	81	2011	wound	USA	CM003318
pRAY*-v1	C2	6078	2	2007	nr	Australia	JF343536
p3ZQ2	ZQ2	6078	2	2016	sputum	Iraq	CM009648
pABLAC2	LAC-4	6076	10	1997	HO [a]	USA	CP007714
pD46-1	D46	6078	25	2010	UTI [b]	Australia	CP048132
pNaval18-6.1	Naval-18	6078	25	2006	nr	USA	AFDA02 [c]
pR32_3	Nord4-2	11378	25	2018	nr	Germany	CP091597
p6ACN21	ACN21	9909	85	2018	blood	India	CP038647
p4ACN21	ACN21	7396	85	2018	blood	India	CP038649
p3ACN21	ACN21	6944	85	2018	blood	India	CP038650
p2ACN21	ACN21	5844	85	2018	blood	India	CP038651
p1ACN21	ACN21	5734	85	2018	blood	India	CP038652
pAbBAS-1.2	AbBAS-1	6078	85	2019	clinical	Spain	CP065394
pMRSN4106-6	MRSN 4106	6078	94	2011	wound	USA	CM003315
pMRSN3942-6	MRSN 3942	6078	94	2011	wound	USA	CM003319
pMRSN3405-6	MRSN 3405	6078	94	2011	wound	USA	CM003320
pJ9-1	J9	6078	49	1999	clinical	Australia	CP041588
p1AR_0070	AR_0070	6078	32	nr	clinical	USA	CP027181
p1AR_0052	AR_0052	6078	32	nr	clinical	USA	CP027187
p2ZQ10	ZQ10	6078	575	2016	CSF [d]	Iraq	CM009031
p3ZQ9	ZQ9	6133	575	2016	blood	Iraq	CM009085
p1ZQ3	ZQ3	6078	717	2016	blood	Iraq	CM009028
p2ZQ8	ZQ8	6078	513	2016	blood	Iraq	CM009034
p1FDAARGOS_533	FDAARGOS_533	6078	57	2016	sputum	USA	CP033770
pRAY*-v2	E7	8433	novel	2008	blood	Australia	JX076770
pMC23.3	MC23	6078	novel	2016	urine	Bolivia	MK531539

[a] hospital outbreak – site not recorded.
[b] urinary tract infection (UTS)
[c] complete GenBank accession number AFDA02000006
[d] cerebrospinal fluid (CSF)

Supplementary Table 4. Distribution of antimicrobial resistance genes in large conjugative plasmid related to pA297-3

Plasmid name	Length (bp)	ST	Year	Isolation source	Country	Antimicrobial resistance genes	Accession number
pA297-3	200633	1	1984	UTI	Netherlands	*sul2, strAB*	KU744946
pOIFC137-122	122461	3	2003	nr [a]	USA	*strAB, sul2*	AFDK01000004
pOIFC109-122	122469	3	2003	nr	USA	*strAB, sul2*	ALAL01000013
pAB3	148955	437	2014	clinical	Canada	*sul2*	CP012005
pAB04-1	169023	10	2012	blood	Canada	*aph(3'')-Ib, strAB, sul2, arr-2, cmlA5,* $bla_{\text{PER-7}}$*, sul1, armA, mph-msr(E), tetB*	CP012007
pPM193665_1	150385	10	2019	Pus	India	*mph-msr(E), armA, sul1, cmlA5, arr-2, sul2, strAB,* ble_{MBL}, bla_{NDM}*, tetB*	CP050416
pPM194122_1	150385	10	2019	BAL [b]	India	*mph-msr(E), armA, sul1, cmlA5, arr-2, sul2, strAB,* ble_{MBL}, bla_{NDM}*, tetB*	CP050426
pOIFC143-128	127633	25	2003	nr	USA	*strAB, sul2*	AFDL01000008
pD4	132632	25	2006	wound	Australia	*sul2, strAB*	CP048851
pD46-4	208004	25	2010	UTI	Australia	*tetB, sul2, mph-msr(E), strAB*	CP048135
p40288	145711	25	2015	UTI	France	-	CP077802
pR32_1	117234	25	2018	nr	Germany	-	CP091598
pCL107	198716	25	2012	UTI	Lebanon	*sul2, tetB, strAB, aacC2*	CP098522
pNaval18-131	130660	25	2006	nr	USA	*sul2, strAB*	AFDA02000009
pHWBA8_1	195838	25	2013	sputum	Korea	*armA, mph-msr(E), tetB, aac(6')-lan, aac(3)-lle, sul2, arr-2, cmlA5, sul1,* $bla_{\text{PER-7}}$	CP020596
pAba7804b	170420	25	2006	BAL	Mexico	*sul2, tetB, strAB*	CP022285
p2AR_0088	146698	25	nr	clinical	USA	*sul2, tetB, strAB, aac(3)-lle, aac(6')-lan*	CP027531
p3P7774	202283	25	2018	Pus	India	*tetB, mph-msr(E), armA,* $bla_{\text{PER-7}}$*, sul1, cmlA5, arr-2, sul2, strAB, aac(6')-lan*	CP040260
pVB82_1	215278	25	2019	blood	India	*aac(6')-lan,* $bla_{\text{OXA-23}}$*, strAB, sul2, tetB, mph-msr(E), armA, sul1,* $bla_{\text{PER-7}}$*, cmlA5, arr-2*	CP050386
p2VB16141	189343	622	2019	blood	India	*strAB, sul2, arr-2, cmlA5,* $bla_{\text{PER-7}}$*, sul1, armA, mph-msr(E)*	CP040051
pIOMTU433	189354	622	2013	clinical	Nepal	*mph-msr(E), armA,* $bla_{\text{PER-7}}$*, sul1, cmlA5, arr-2, sul2, strAB*	AP014650
p1KSK6	218105	622	2020	respiratory	India	*mph-msr(E), armA,* $bla_{\text{PER-7}}$*, sul1, cmlA1, arr-3, ant(3'')-la, sul2, strAB,*	CP072271
p1KSK7	218105	622	2020	respiratory	India	*mph-msr(E), armA,* $bla_{\text{PER-7}}$*, sul1, cmlA1, arr-3, ant(3'')-la, sul2, strAB,*	CP072276

p1KSK10	218105	622	2020	respiratory	India	*mph-msr(E), armA, bla*$_{\text{PER-7}}$*, sul1, cmlA1, arr-3, ant(3")-la, sul2, strAB,*	CP072281
p1KSK11	218105	622	2020	respiratory	India	*mph-msr(E), armA, bla*$_{\text{PER-7}}$*, sul1, cmlA1, arr-3, ant(3")-la, sul2, strAB,*	CP072286
p1KSK18	218105	622	2020	respiratory	India	*mph-msr(E), armA, bla*$_{\text{PER-7}}$*, sul1, cmlA1, arr-3, ant(3")-la, sul2, strAB,*	CP072291
p1KSK19	218105	622	2020	respiratory	India	*mph-msr(E), armA, bla*$_{\text{PER-7}}$*, sul1, cmlA1, arr-3, ant(3")-la, sul2, strAB,*	CP072296
p1KSK20	218105	622	2020	respiratory	India	*mph-msr(E), armA, bla*$_{\text{PER-7}}$*, sul1, cmlA1, arr-3, ant(3")-la, sul2, strAB,*	CP072301
p1KSK2	218105	622	2020	respiratory	India	*mph-msr(E), armA, bla*$_{\text{PER-7}}$*, sul1, cmlA1, arr-3, ant(3")-la, sul2, strAB,*	CP072399
pNCTC7364	148956	494	2014	nr	UK	*sul2*	LT605060
pB11911	216780	149	2014	blood	India	*strAB, sul2, arr-2, cmlA5, bla*$_{\text{PER-7}}$*, sul1, armA, mph-msr(E)*	CP021344
p3VB35179	236166	1512	2018	blood	India	*strAB, sul2, arr-2, cmlA5, bla*$_{\text{PER-7}}$*, sul1, armA, mph-msr(E), tetB*	CP040054
pA1429c	205113	108	2010	secretion	China	*bla*$_{\text{TEM-1}}$*, aac(3)-lle, aac(6')-lan, strAB, tetB, sul2*	CP046899
pPM194229_1	226394	447	2019	BAL	India	*mph-msr(E), armA, sul1, bla*$_{\text{PER-7}}$*, cmlA5, arr-2, strAB, sul2, tetB*	CP050433
p1KSK1	218105	865	2020	respiratory	India	*mph-msr(E), armA, sul1, bla*$_{\text{PER-7}}$*, cmlA1, arr-3, ant(3")-la, strAB,*	CP072123
pAb45063_b	183767	nk	nr	nr	Brazil	*sul2, strAB*	MK323043
pMC1.1	184770	nk	2015	catheter	Bolivia	*aacC5, aac(6')-lan, strAB, tetB, sul2*	MK531536
pMC75.1	150158	nk	2016	ulcer	Bolivia	*strA, aph(6), sul2*	MK531540

[a] not recorded

[a] Broncho-alveolar lavage (BAL)

Supplementary Table 5. Properties of plasmids encoding the MPF_T transfer system that carry *bla*$_{NDM}$

Plasmid name	Length (bp)	Isolation source	Country	Year	ST	Antimicrobial resistance genes	Accession number
pOCU_Ac16a_2	41087	tracheal aspirate	Japan	2015	412	*bla*$_{NDM-1}$, *bleMBL, aphA6*	AP023079
p6200-47.274kb	47274	bodily fluid	Colombia	2012	464	*bla*$_{NDM-1}$, *bleMBL, aphA6*	CP010399
pNDM-0285	39359	wastewater	USA	2016	1543	*bla*$_{NDM-1}$, *bleMBL, aphA6*	CP026127
p1AR_0088	41087	clinical	USA	<2013	25	*bla*$_{NDM-1}$, *bleMBL, aphA6*	CP027532
pAbNDM-1	48368	feces	China	<2013	639	*bla*$_{NDM-1}$, *bleMBL, aphA6*	JN377410
pNDM-AB	47098	pig lung	China	<2013	novel	*bla*$_{NDM-1}$, *aphA6, mph-msr(E)*	KC503911
Piec383	47283	blood	Brazil	2014	novel	*bla*$_{NDM-1}$	MK053932
pAB17	41087	-	Brazil	<2020	novel	*bla*$_{NDM-1}$, *aphA6*	MT002974

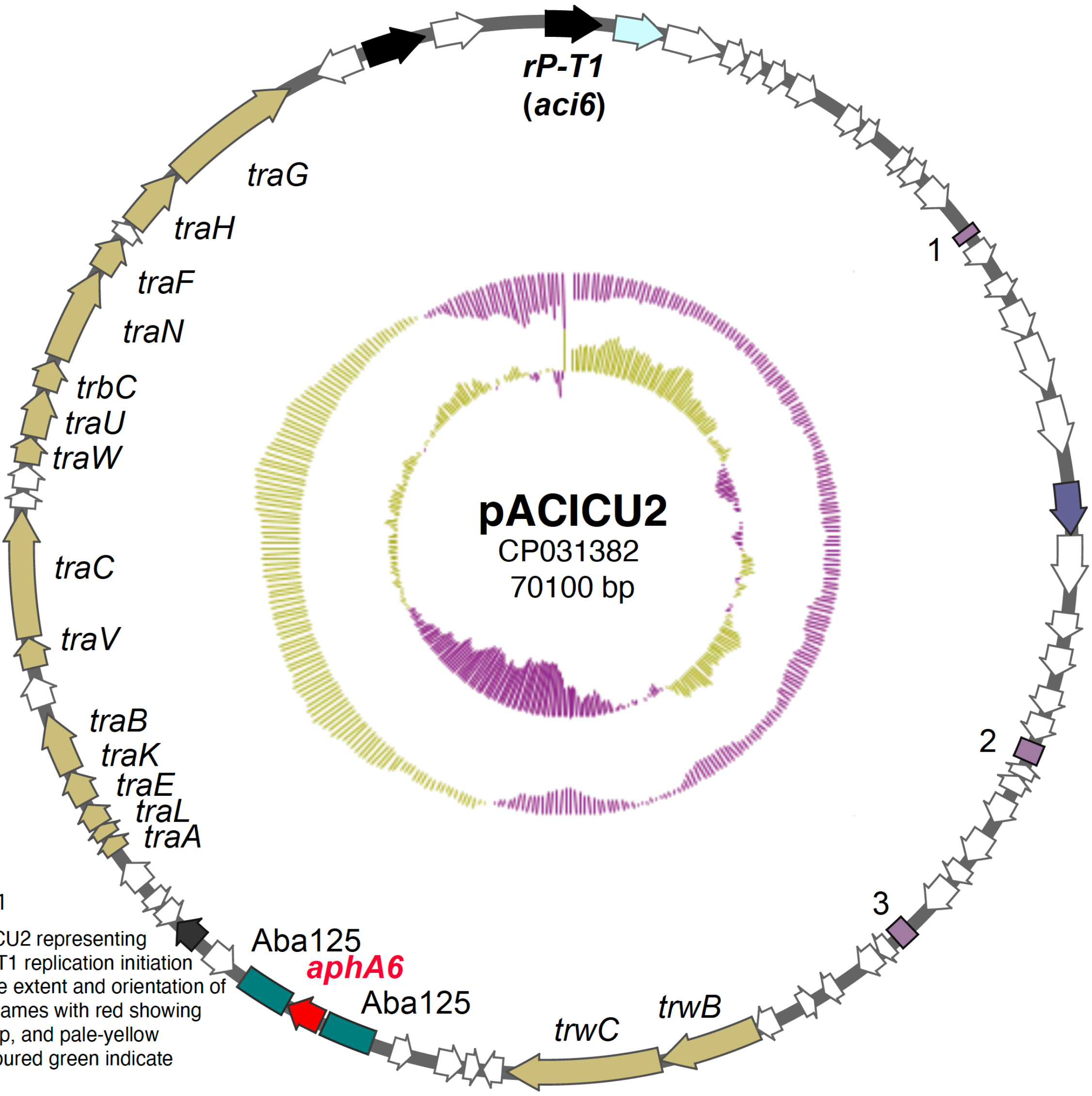

Supplementary Figure 1

Genetic structure of pACICU2 representing plasmids that encode RP-T1 replication initiation protein. Arrows indicate the extent and orientation of genes and open reading frames with red showing resistance genes, black rep, and pale-yellow transfer genes. Boxes coloured green indicate ISAba125 copies.

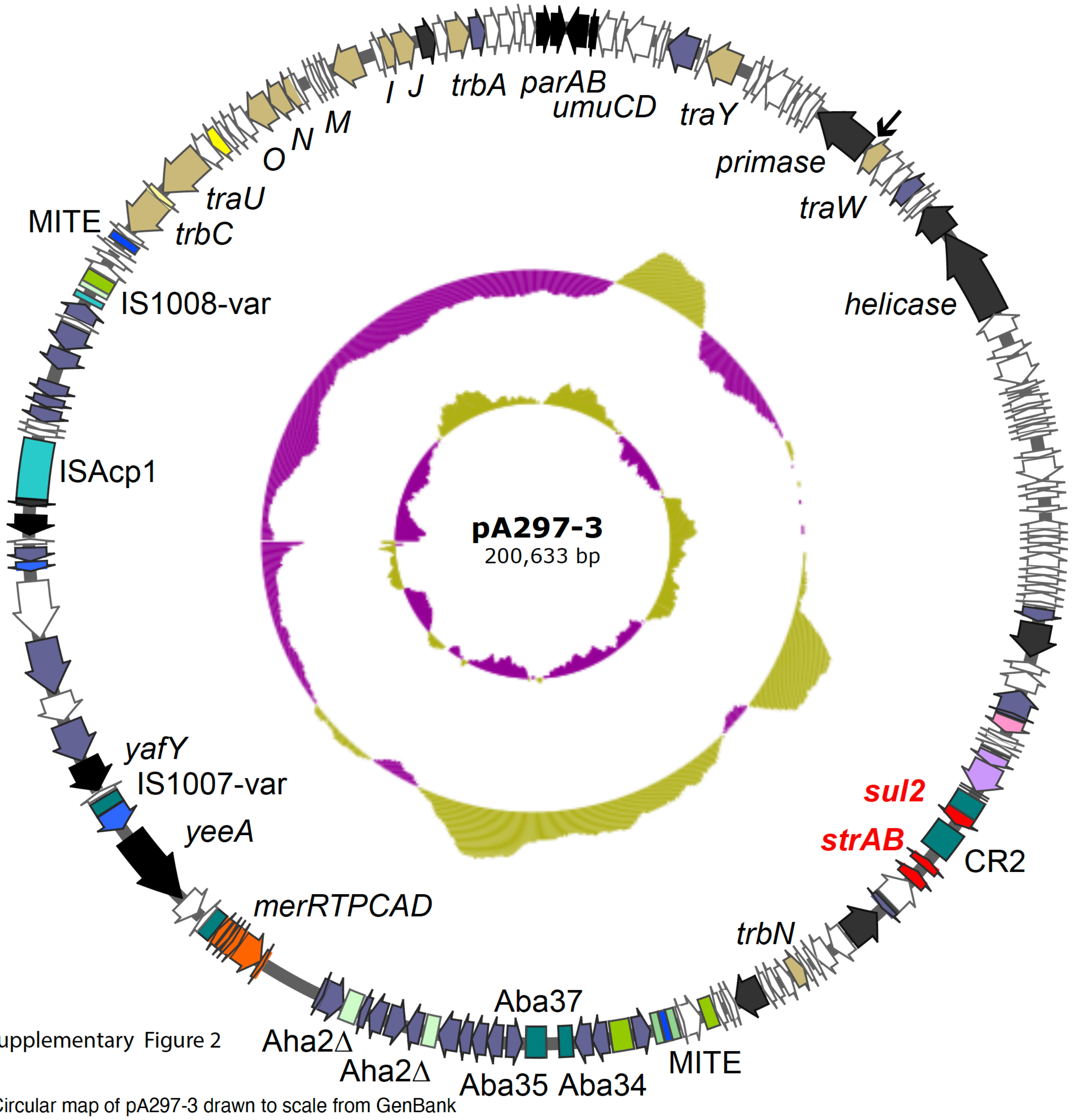

Supplementary Figure 2

Circular map of pA297-3 drawn to scale from GenBank accession number KU744946. Arrows represent the orientation and extent of genes and open reading frames. Open reading frames with no predicted function are white and antimicrobial resistance genes are coloured red. Insertion sequences (IS) are shown with filled boxes coloured different shades of green. Arrows coloured pale-yellow represent the tra genes, which are involved in plasmid transfer. Gray arrows represent genes/orfs involved in DNA metabolism.
