# Supplementary material for: Examining the role of Acinetobacter baumannii plasmid types in disseminating antimicrobial resistance
Source: NPJ Antimicrob Resist. 2024 Jan 5;2:1. doi: 10.1038/s44259-023-00019-y (PMC11702686; doi:10.1038/s44259-023-00019-y)
Supplement: Supplementary file 2 — Reporting Summary [file 44259_2023_19_MOESM2_ESM.pdf]

nature portfolio

Corresponding author(s): Mehrad Hamidian

Last updated by author(s): Nov 13, 2023

Reporting Summary

Nature Portfolio wishes to improve the reproducibility of the work that we publish. This form provides structure for consistency and transparency in reporting. For further information on Nature Portfolio policies, see our Editorial Policies and the Editorial Policy Checklist.

Statistics

For all statistical analyses, confirm that the following items are present in the figure legend, table legend, main text, or Methods section.

n/a	Confirmed	
☐	☒	The exact sample size (*n*) for each experimental group/condition, given as a discrete number and unit of measurement
☒	☐	A statement on whether measurements were taken from distinct samples or whether the same sample was measured repeatedly
☒	☐	The statistical test(s) used AND whether they are one- or two-sided<br>*Only common tests should be described solely by name; describe more complex techniques in the Methods section.*
☒	☐	A description of all covariates tested
☒	☐	A description of any assumptions or corrections, such as tests of normality and adjustment for multiple comparisons
☒	☐	A full description of the statistical parameters including central tendency (e.g. means) or other basic estimates (e.g. regression coefficient) AND variation (e.g. standard deviation) or associated estimates of uncertainty (e.g. confidence intervals)
☒	☐	For null hypothesis testing, the test statistic (e.g. *F*, *t*, *r*) with confidence intervals, effect sizes, degrees of freedom and *P* value noted<br>*Give P values as exact values whenever suitable.*
☒	☐	For Bayesian analysis, information on the choice of priors and Markov chain Monte Carlo settings
☒	☐	For hierarchical and complex designs, identification of the appropriate level for tests and full reporting of outcomes
☒	☐	Estimates of effect sizes (e.g. Cohen's *d*, Pearson's *r*), indicating how they were calculated

Our web collection on statistics for biologists contains articles on many of the points above.

Software and code

Policy information about availability of computer code

Data collection	A local database of complete A. baumannii plasmids that were publicly available as of mid-August 2022 was generated. Our local database included plasmid sequences of i) 354 complete genomes out of 449 complete A. baumannii genomes (i.e. n=95 entries with no plasmids) sourced from GenBank (https://www.ncbi.nlm.nih.gov/genome/browse/#!/prokaryotes/403/); labelled as 'Complete genome project' as data source in Table S1 and Table S2) and ii) an additional 92 genomes/unique strains (released between February 2021 and mid-August 2022) captured in RefSeq (https://www.ncbi.nlm.nih.gov/refseq/). The latter included n=29 genomes sourced from Whole Genome Shotgun projects (labelled as 'WGS' in Table S2) and n=63 unique strains that were not linked to a genome project (i.e. direct plasmid submission to GenBank; labelled as 'GenBank non-redundant db' in Table S2). This resulted in the curation of our final dataset consisted of 813 non-redundant plasmid entries corresponding to at least 439 unique isolates (n=354 isolates from Complete genome projects, n=63 from GenBank non-redundant database, and n=29 from WGS). Of the 813 plasmid entries, n=620 were those we previously used to develop the Acinetobacter Plasmid Typing scheme 13. Note that n=621 plasmids had been included in the original scheme, but n=1 plasmid (accession CP059478.1) was excluded from this current study as it was found to be associated with a non-baumannii genome (i.e. A. pittii). All supporting data and protocols have been provided within the article or through supplementary data files. The online version of this article has four supplementary tables and three supplementary figures.
Data analysis	The chromosomal sequences associated with each plasmid were found by exporting the BioSample accession numbers using the RefSeq https://www.ncbi.nlm.nih.gov/refseq/ followed by the curation of a list of chromosomal GenBank accession numbers and downloading the sequence data through Entrez Programming Utilities (E-utilities; https://www.ncbi.nlm.nih.gov/books/NBK25501/). The chromosomal sequences were uploaded to Pathogenwatch (https://pathogen.watch/) and the Speciator tool was used to verify that the sequences were A. baumannii. Multi-locus Sequence Types (MLSTs) were assigned using the mlst v.2.0 software (https://github.com/tseemann/mlst). Standalone BLAST (https://ftp.ncbi.nlm.nih.gov/blast/executables/LATEST/) was used for plasmid sequence comparisons within the rep-less plasmid group and assign 'related known plasmid' variants as labelled in Table S2. The SnapGene® (V.6.0.5) software was used to examine the

structure of individual plasmids. The plasmids were screened for AMR genes using Abricate v1.0.1 (available at https://github.com/tseemann/abricate) using the ResFinder v.2.1 database (available under https://cge.cbs.dtu.dk/services/ResFinder/). Data visualisation was performed using the ggplot2 package (https://ggplot2.tidyverse.org/) in R (v1.1.456) and Adobe Illustrator (V23.0.3).

For manuscripts utilizing custom algorithms or software that are central to the research but not yet described in published literature, software must be made available to editors and reviewers. We strongly encourage code deposition in a community repository (e.g. GitHub). See the Nature Portfolio guidelines for submitting code & software for further information.

Data

Policy information about availability of data

All manuscripts must include a data availability statement. This statement should provide the following information, where applicable:

- Accession codes, unique identifiers, or web links for publicly available datasets
- A description of any restrictions on data availability
- For clinical datasets or third party data, please ensure that the statement adheres to our policy

All plasmid sequence data analysed in this study is publicly available in the RefSeq (https://www.ncbi.nlm.nih.gov/refseq/) database in GenBank with GenBank/RefSeq accession numbers provided in Supplementary Tables 1 and 2. All curated Rep/rep sequences are available under https://github.com/MehradHamidian/AcinetobacterPlasmidTyping.

Research involving human participants, their data, or biological material

Policy information about studies with human participants or human data. See also policy information about sex, gender (identity/presentation), and sexual orientation and race, ethnicity and racism.

Reporting on sex and gender	Not applicable
Reporting on race, ethnicity, or other socially relevant groupings	Not applicable
Population characteristics	Not applicable
Recruitment	Not applicable
Ethics oversight	Not applicable

Note that full information on the approval of the study protocol must also be provided in the manuscript.

Field-specific reporting

Please select the one below that is the best fit for your research. If you are not sure, read the appropriate sections before making your selection.

☒ Life sciences ☐ Behavioural & social sciences ☐ Ecological, evolutionary & environmental sciences

For a reference copy of the document with all sections, see nature.com/documents/nr-reporting-summary-flat.pdf

Life sciences study design

All studies must disclose on these points even when the disclosure is negative.

Sample size	Sequence data collected for this study included all complete A. baumannii plasmids that were publicly available as of mid-August 2022 was generated. Our local database included plasmid sequences of i) 354 complete genomes out of 449 complete A. baumannii genomes (i.e. n=95 entries with no plasmids) sourced from GenBank (https://www.ncbi.nlm.nih.gov/genome/browse/#!/prokaryotes/403/); labelled as 'Complete genome project' as data source in Table S1 and Table S2) and ii) an additional 92 genomes/unique strains (released between February 2021 and mid-August 2022) captured in RefSeq (https://www.ncbi.nlm.nih.gov/refseq/).
Data exclusions	All sequence data with poor quality were excluded (provided as supplementary material)
Replication	Not applicable
Randomization	Not applicable
Blinding	Not applicable

Reporting for specific materials, systems and methods

We require information from authors about some types of materials, experimental systems and methods used in many studies. Here, indicate whether each material, system or method listed is relevant to your study. If you are not sure if a list item applies to your research, read the appropriate section before selecting a response.

Materials & experimental systems

n/a	Involved in the study
☒	☐ Antibodies
☒	☐ Eukaryotic cell lines
☒	☐ Palaeontology and archaeology
☒	☐ Animals and other organisms
☒	☐ Clinical data
☒	☐ Dual use research of concern
☒	☐ Plants

Methods

n/a	Involved in the study
☒	☐ ChIP-seq
☒	☐ Flow cytometry
☒	☐ MRI-based neuroimaging

Plants

Seed stocks	Not applicable
Novel plant genotypes	Not applicable
Authentication	Not applicable
